# Detected residual venous thrombi and catheter-directed management of intermediate-risk pulmonary thromboembolism

**DOI:** 10.3389/fcvm.2026.1837276

**Published:** 2026-06-05

**Authors:** Samuel N. Heyman, David Leibowitz, Eyal Herzog

**Affiliations:** Departments of Medicine and Cardiology, Hadassah Hospitals and the Hebrew University Medical School, Jerusalem, Israel

**Keywords:** ambulation, deep vein thrombosis, Doppler ultrasound, embolectomy, pulmonary hypertension, pulmonary thromboembolism, thrombolysis

## Abstract

Retained clots leading to recurrent episodes of thromboembolism are a major cause of early mortality post PE, that may reach 17% within 3 months after the first episode. The use of catheter-directed interventions in the management of pulmonary embolism is currently restricted to hemodynamically unstable patients and to those classified as intermediate-high risk individuals, based on cardiac echo and troponin. Current guidelines do not recommend these strategies in patients classified as intermediate-low risk (i.e., evidence of RV compromise by echo but without elevated troponin). We propose a mandatory evaluation of the presence or absence of retained clots within great veins, particularly in this subset of patients, since detecting retained large ileo-femoral thrombi (that are identified in some 50% of patients) may affect treatment decisions, favoring catheter-directed interventions, as relieved RV afterload may enable coping with repeated PE episodes. Our hypothesis requires validation in prospective clinical trials.

## Introduction

**Figure 1:**
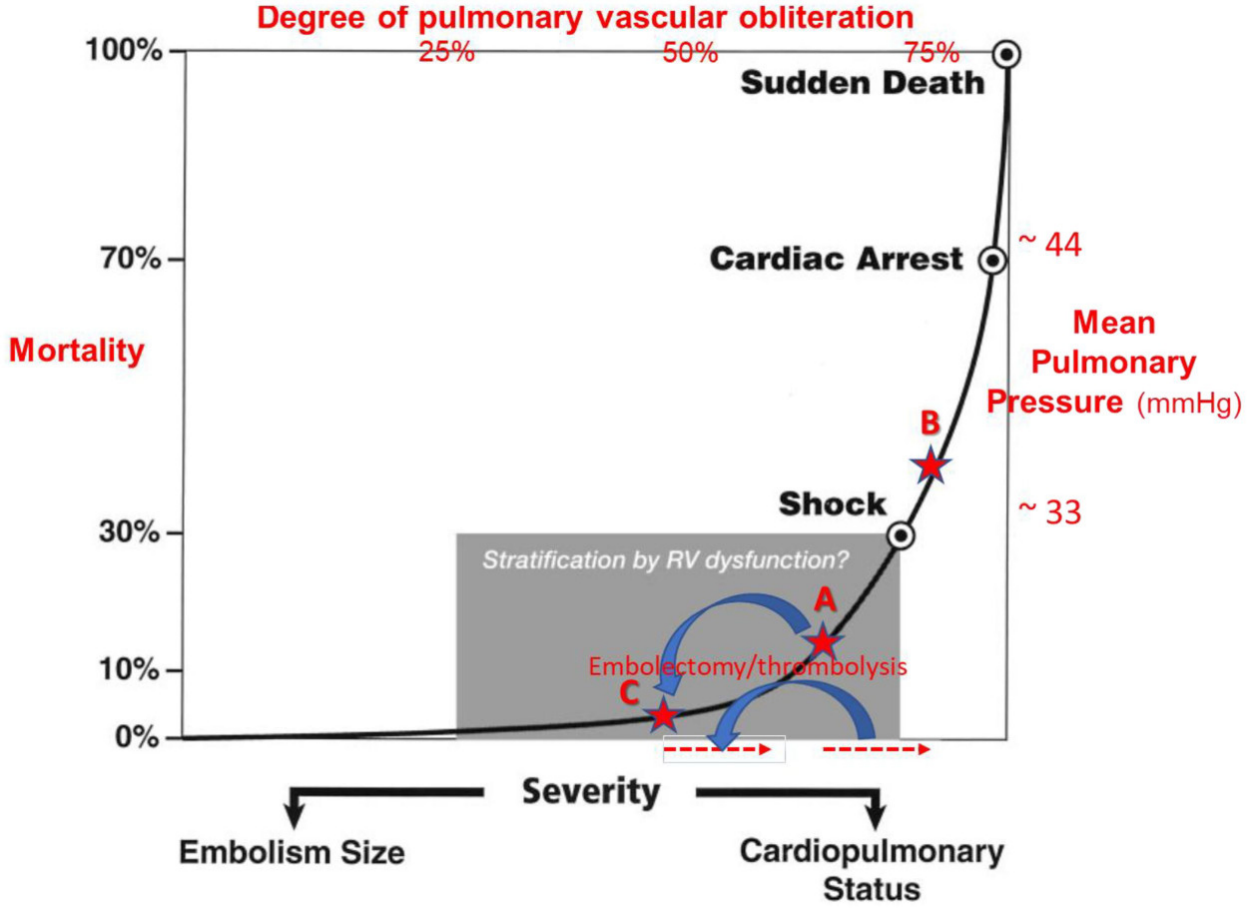
A schematic presentation of the association between loss of pulmonary vasculature patency and rising pulmonary pressure. This low resistance vascular system can tolerate some 50% vascular obliteration by massive emboli without a significant increase in pulmonary pressure. But beyond this point, pulmonary pressure rises exponentially by even small additional embolic episodes (from A to B), with consequent hemodynamic instability (obstructive shock) and the development of pulseless electrical activity and death. Reducing RV afterload by catheter-directed interventions (embolectomy and/or thrombolysis) will move the hemodynamic status to the left along the declining curve (upper blue arrow from A to C), in a way that a repeated comparable embolic episode (dashed red arrows at the bottom) will be tolerable (lower blue arrow) and not associated with a life-threatening rise in pulmonary pressure. Compromised cardiopulmonary status related to other co-morbidities might also be associated with a much earlier rising slope of pulmonary pressure and lethality, generated by rather small emboli. [modified from Wood KE, Chest 2002 (3)].

Catheter-directed interventions, such as thrombolysis, embolectomy or their combination have become a principal therapeutic option in the management of patients with life-threatening pulmonary emboli (PE) (1), shifting the pulmonary hemodynamic status curve depicted in Figure 1 to the left, relieving critical pulmonary hypertension. Yet, its usage is currently restricted by guidelines to those hemodynamically unstable patients and to those categorized as intermediate-high-risk PE [i.e., patients with substantially increased afterload with compromised Rt. ventricular (RV) function or with a high Pulmonary Emboli Severity Index (PESI) score, combined with increased plasma troponin, with or without elevated BNP/NT-pro-BNP] (2). Individuals with compromised RV but without elevated enzymes are defined as intermediate low-risk PE patients, where systemic thrombolysis or catheter-directed interventions are currently not recommended. Herein we propose to consider extending the indications for catheter-directed interventions, including intermediate low-risk patients with retained large above-knee venous clots. Our proposal is based on the association between such retained thrombi with increased mortality and on the physiologic rationale, that reducing RV afterload will enable withstanding repeated potentially lethal thromboembolic events.

### Physiologic perspectives

Fatality in PE principally results from critically increased pulmonary pressure, leading to acute RV failure and hemodynamic instability that evolves to pulseless electrical activity. The extent of obliteration of the pulmonary vasculature and baseline pulmonary pressure are the principal parameters governing this scenario (3). As shown in Figure 1, the low-resistant pulmonary vasculature can tolerate a substantial diminution in the vascular cross-sectional area without an overt increase in RV afterload. Nevertheless, obliteration by emboli of more than 40%–60% of the pulmonary vascular bed generates pulmonary hypertension that increases exponentially as the extent of thromboembolism increases. This implies that even small recurrent emboli at this point may suffice to generate a fatal outcome. Chronic pulmonary hypertension, including preceding chronic organized emboli can lower this threshold and generate life-threatening pulmonary hypertension by a small burden of recurrent emboli.

However, current guidelines do not address the possibility that a compromised RV performance might be dramatically hampered by an additional thromboembolic event, even a small one, if the hemodynamic status is located at the rising slope depicted in Figure 1. For that reason, the presence of additional “thrombus burden”, i.e., retained clots within the venous system might be detrimental, favoring strategies to relieve pulmonary hypertension in order to reduce the risk of fatal outcome with additional embolic events. Conceivably, the larger the retained clot—the higher is the risk for a recurrent potentially fatal event. Therefore, the proximity of the venous clot should be considered, with likely ominous potential especially for popliteal and ileo-femoral DVT.

### Retained venous clots predict mortality following an initial thromboembolic event

Early mortality following massive PE is substantial (4), reaching 12.5% and 16% by 30 and 90 days, respectively, despite the initiation of anticoagulant therapy. Only seventeen percent, of these patients underwent thrombolysis, perhaps implying that persistent pulmonary hypertension underlies the susceptibility to lethal events upon repeated embolic events. In line with this possibility are reports linking documented retained venous thrombi with the risk of death during convalescence from PE, despite anticoagulation. Jiménez et al. (5) conducted a large single center prospective study over 4 years, assessing the impact of residual deep vein thrombi (DVT) on mortality in patients recovering from PE. Comparing outcomes of 362 patients with DVT as documented by compression ultrasound, with 345 DVT-free patients, they reported doubling of the risk of death at 90 days among patients with retained venous thrombi, with a 4.25 hazard ratio of PE-related death (5). Furthermore, in patients with PE and RV dysfunction, the presence of concomitant DVT was associated with a 19.6% 30-day mortality rate (19.6%), a better predictive value than the presence of elevated troponin (15.2%) (6). In an extended multinational study, including 591 normotensive patients with PE, retained venous clots were highly predictive of 30-day PE-related mortality indicating that documented retained venous clots was the 3rd statistically significant predictive factor (OR 2.66), preceded only by cardiac troponin (OR 2.66) and RV dysfunction (OR 2.57) (7). A meta-analysis including 7,868 patients with acute symptomatic PE, 4,379 of them with concomitant lower extremity DVT. showed that the presence of retained venous clots was associated with increased 30-day mortality (OR 1.89) (8). Finally, an analysis of the RIETE Registry of 17,742 patients with symptomatic PE and concomitant documented lower limb DVT, revealed that the presence of symptomatic DVT predicted 30-day all-cause death (adjusted hazard ratio 1.49) and PE-related mortality (OR 1.52) (9).

## Discussion

Collectively, these outcomes indicate a close association between retained DVT with 30–90-day mortality in patients with symptomatic PE. It is tempting to assume that incomplete lysis of clots within the pulmonary vasculature following an initial event, associated with pulmonary hypertension, predisposed to mortality caused by recurrent thromboembolism, and that the resolution of these initial emboli, relieving substantial pulmonary hypertension and RV dysfunction, might improve prognosis during subsequent embolic events. We have therefore suggested a modification of the current guidelines regarding catheter-directed procedures, in intermediate-low risk patients where these procedures are currently not recommended (10). We propose to routinely and mandatorily evaluate the presence of residual thrombi within the lower extremities in intermediate low-risk patients showing RV compromise. We argue that the presence of coexisting DVT should be taken into account in such patients, favoring interventions to reverse pulmonary hypertension, relieving RV dysfunction to enable it handling repeated episodes of PE. Jiménez et al. reached similar conclusions over 15 years ago (6), yet, at that time the use of catheter-directed interventions has been limited, and systemic thrombolysis was an unfavorable option due to potential life-threatening hemorrhagic complications. Currently, these procedures have gained popularity as recent meta-analyses and systematic reviews suggest that catheter-directed thrombolysis is associated with a substantially lower mortality compared to anticoagulation alone in intermediate-risk PE, but with a similar or only slightly increased risk of major bleeding (11). In this respect, the recent introduction of Ultrasound-Accelerated Thrombolysis (USAT) shows promising efficacy in reducing RV afterload with a favorable safety profile (12). With that in mind, the presence of retained popliteal or above-knee thrombi in such intermediate-risk individuals, anticipated to be detected is about 50% of patients (5), might select those who would especially benefit from catheter-directed interventions. If the patient deteriorates hemodynamically despite the suggested intervention, or wile applying it, the use of extracorporal membrane oxygenation (ECMO) as a rescue tool is warranted, as outlined in the algorithm by Baran et al. (13).

Catheter-directed thrombolysis and mechanical thrombectomy are being evaluated in ongoing trials (HI-PEITHO, PE-TRACT, PEITHO-3, PEERLESS), but to our knowledge these studies do not specifically target patients with concomitant large above-knee or symptomatic DVT. The American Heart Association scientific statement highlights the need for enriched trial populations with additional markers of severity, but unfortunately does not address large or symptomatic DVT as a pre-specified subgroup (14).

Prospective clinical studies indicate an advantage for early ambulation following PE. Yet, hemodynamically unstable patients were excluded from such works, and such individuals may be particularly at risk of lethal repeated embolic events. Thus, routine duplex studies may also affect decisions regarding early ambulation in intermediate risk patients with RV dysfunction, particularly if the option of catheter-directed intervention is deferred or not recommended by current guidelines. Under such circumstances, detection of retained proximal (popliteal or ileo-femoral) venous clots in a patient with an already compromised RV function, may prompt catheter-directed intervention. The insertion of a temporary vena cava filter before ambulation in such cases may be considered only if thrombolysis and full anticoagulation are contraindicated and debulking of the pulmonary obliterative process by means of embolectomy is impractical. Noteworthy, to our knowledge, the risk of death related to repeated PE during early ambulation in this particular subgroup of patients with RV compromise has not been explored, so far and is also yet to be defined.

**Figure 2:**
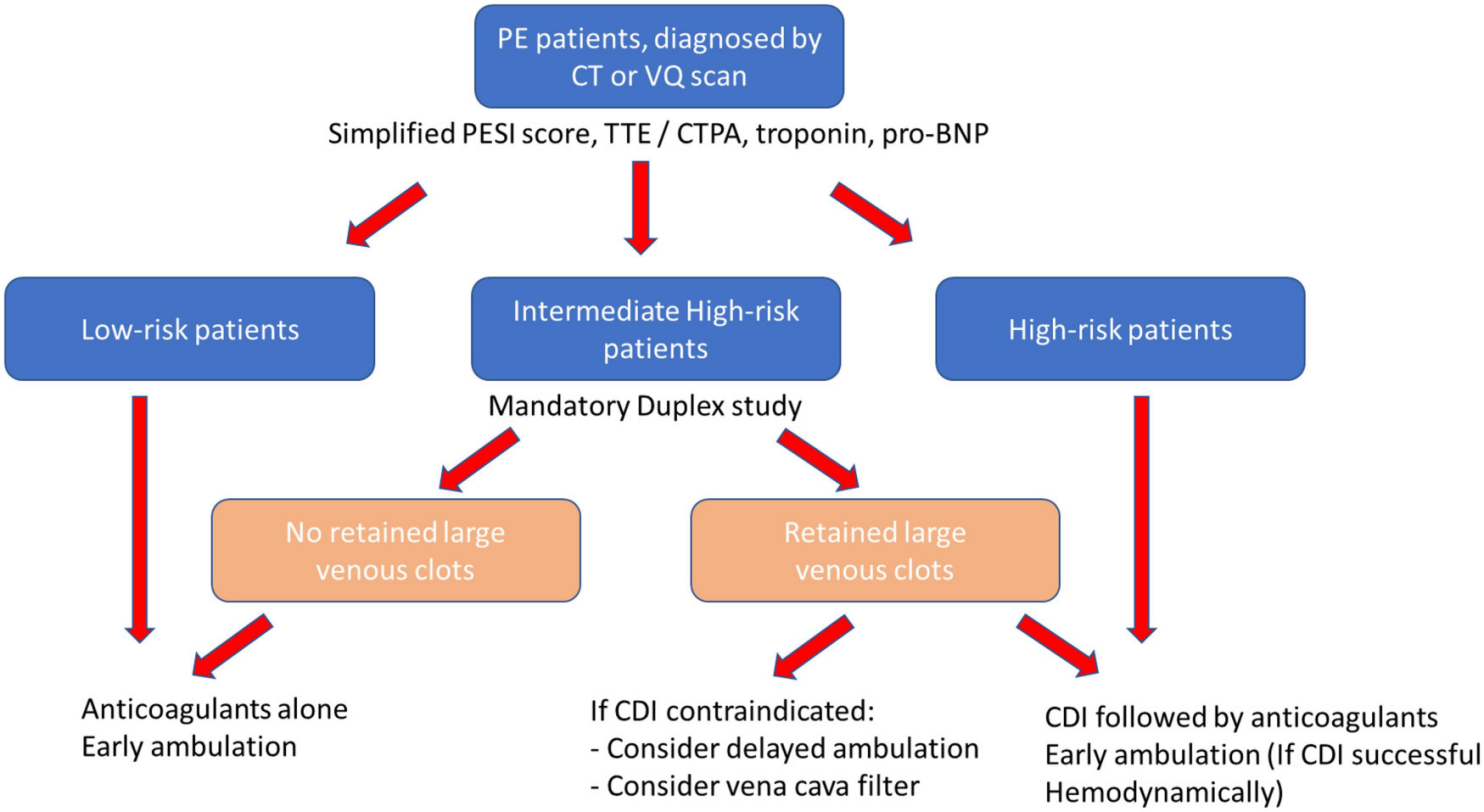
A proposed algorithm showing the assessment and management of hemodynamically stable individuals with PE, focusing on intermediate-low-risk patents. This category is defined as evident Rt. ventricular compromise (RV dilatation, reduced RV systolic function, marked pulmonary hypertension, lack of vena cava collapse on inspiration) or as a high simplified PE score index (PESI), in the absence of rising troponin and/or pro-BNP (10). A mandatory duplex study in such patients should help identify those with retained above-knee large venous clots, susceptible to repeated potentially lethal thromboembolic events. Catheter-directed intervention (CDI) should be considered in this case, along with decisions regarding delaying ambulation.

In conclusion, as outlined in our previous report (10), and in Figure 2, an algorithm focusing on intermediate-low-risk patients with PE, we propose to routinely perform duplex studies in patients with intermediate-low risk PE. Patients with documented retained large (popliteal or ileo-femoral) clots should be considered as high-risk patients, prone to potentially lethal repeated thromboembolism, particularly upon early mobilization. Catheter-directed interventions may reduce augmented RV afterload and the lethal potential of a repeated thromboembolic episode. Such patients should be considered a prespecified subgroup in future prospective studies examining the role of catheter directed therapy.

## Data Availability

Publicly available datasets were analyzed in this study. This data can be found here: no data base was used.
